# Intellectual Disability and Parenthood

**DOI:** 10.1100/tsw.2005.12

**Published:** 2005-01-21

**Authors:** Isack Kandel, Mohammed Morad, Gideon Vardi, Joav Merrick

**Affiliations:** ^1^ Faculty of Social Science, Department of Behavioral Sciences, Academic College of Judea and Samaria, Ariel, DN Ephraim 44837, Israel; ^2^ Division of Community Health, Ben Gurion University, Beer-Sheva, Israel; ^3^ Zusman Child Development Center, Division of Pediatrics, Ben Gurion University, Beer-Sheva, Israel; ^4^ National Institute of Child Health and Human Development, Office of the Medical Director, Division for Mental Retardation, Ministry of Social Affairs, Jerusalem and Division of Pediatrics, Ben Gurion University, Beer-Sheva, Israel

**Keywords:** mental retardation, developmental disability, intellectual disability, human development, child health, public health, Israel

## Abstract

Parenthood in persons with intellectual disability (ID) is an issue of concern for the family, guardians, and professionals as there are many sentiments and problems involved: financial, technical, medical, legal, and above all moral. People with intellectual, developmental, or other disabilities have feelings, want relationships, and are able to have children also. The attitude of society has changed through time from the early eugenic concern with heredity and fertility, to a focus on the risk to the children due to parental neglect or abuse, to acceptance and a search for solutions to parental training and support. This change can be seen as a result of a shift from institutional care to community care and normalization. This paper reviews available research, prevalence, service issues, experience from around the world, and relates to the situation in Israel. Jewish Law has been very progressive regarding the possibility of marriage between persons with ID (in contrast to American Law where historically this right has been denied, until recently). Recent research has shown that, in the case of such a union resulting in children, although they require *some* supervision, family, friends, and social welfare agencies have scrutinized these families so much they are in constant fear of their child being taken away. There is little information on the number of such cases and an overall dearth of information on the effects on the children, although one recent study from the U.K. has shown a varied picture of resilience and a close, warm relationship later on with the family and especially the mother.

## INTRODUCTION

Parenthood in persons with intellectual disability (ID) is an issue of concern for the family, guardians, and professionals such as physicians, psychiatrists, psychologists, counselors, social workers, child protection workers, clergy, officials in various institutions, or lawyers. The sentiments and problems involved (financial, technical, medical, legal, and above all moral) when persons with ID contemplate relationships, marriage, and parenthood make it a very difficult task for all involved.

People with intellectual, developmental, or other disabilities have feelings, want relationships, and are able to have children also. The attitude of society has changed through time from the early eugenic concern with heredity and fertility, to a focus on the risk to the children due to parental neglect or abuse, to acceptance and a search for solutions to parental training and support[1]. This change can be seen as a result of a shift from institutional care to community care and normalization[1].

This paper will look at available research, prevalence, service issues, experience from around the world, and relates to the situation in Israel.

## SEXUALITY AND PERSONS WITH DISABILITY

Persons with physical, cognitive, or emotional disabilities should have a right to sexuality education, sexual healthcare, and sexual expression. Family, healthcare workers, and other caregivers should therefore receive training in understanding and supporting sexual development and behavior, comprehensive sexuality education, and related healthcare for individuals with disabilities. The policies and procedures of social agencies and healthcare delivery systems should ensure that services and benefits are provided to all persons without discrimination. Individuals with disabilities and their caregivers should have information and education about how to minimize the risk of sexual abuse and exploitation. People with physical or intellectual disabilities are mostly regarded as nonsexual by society, since sex is associated with youth and physical attractiveness, and not with disability. If we accept that sexual expression is a natural and important part of human life, then the denial of sexuality for disabled persons would be to deny a basic right. There have been many barriers on sexuality over time to this population, both from workers who may be influenced by these views and from disabled people themselves in terms of gaining access to information and acceptance as sexual beings.

In relation to ID, society over time has taken the view that intellectually disabled people have no rights to pursue social and sexual relationships. There has been very little sex education for this population. Intellectually disabled people are sometimes regarded as sexually deviant because they sometimes exhibit socially inappropriate sexual behavior. It is important for educators — particularly those involved in educational programs with disability workers or disabled people — to understand community attitudes towards disability and sexuality, and the impact of these views on disabled people themselves.

## EARLIER STUDIES ON INTELLECTUAL DISABILITY AND PARENTHOOD

In her research into the marriage of persons with ID in the U.K. in the 1950s, Bass[2] observed that women who were sterilized succeeded more in their marriages. Due to this observation, she maintained that there should be a law to enforce the sterilization of women with ID (with their consent and that of the family), especially when there was genetic danger of giving birth to a retarded child.

In their follow-up of the marriage of one couple with ID, Bowden et al.[3] found that marital problems became worse with the birth of a child. A study of 130 persons with ID in Ireland in 1971[4] found that only one-third of the couples were able to care for their children without outside help. Among the children of couples that did receive help, they did not find differences between these children concerning education in comparison with other children in the community and the children growing up in the home functioned better than those in institutions. They maintained that if a couple had children, but did not receive professional help, they would most likely fail. Mattinson[5], from the U.K., examined 32 couples with ID living in residential care before their marriage. Children were born to 17 of these couples and 25% of all these children were placed in residential care due to their own ID.

## PREVALENCE OF PARENTHOOD

Prevalence studies of parents with ID are hard to conduct and information in this area is scarce. There are many reasons for this, some due to the fact that many parents are either not receiving service, some are not identified by the service system, and some are identified, but do not participate in any programs.

Some information can be found from the Arc's Department of Research[6] based on a study done by the Oregon Developmental Disabilities Council in 1989. The study identified 358 families in the state of Oregon with parents considered to have ID. Based on a 1990 Oregon population of 2,853,733, the number of parents with ID would equal 0.00013% of Oregon's general population. If these numbers reflect the general population of the U.S., then 0.00013% of the U.S. population of 249,632,692 in 1990 would equal 32,452 parents with ID, but that is probably a low estimate since many parents are not identified by the service system[6].

One national survey was conducted in Norway[7] in 1997 by sending a questionnaire out to all municipalities for the public health nurses. It showed that 23 persons with ID had given birth within the 12 months before the survey. A total of 126 children with parents with ID were identified, giving an incidence of 27 children per year and a prevalence of about 430 children under 16 years of age in this population of 3.4 million people.

## THE CONCEPT OF MARRIAGE IN JUDAISM

In the modern State of Israel, marriage is an act based on religious law or Halacha (Religious Jewish Law), and the rabbinate is the only established authority sanctioned to perform a marriage ceremony. In Judaism, marriage is the ideal human state of affairs and considered the basic institution established by G-d from the time of creation. In the Bible, it is clearly stated that the purposes of marriage are companionship and creation of the next generation with the following statement for companion: "It is not good that man should be alone, I will make him a help for him….. Therefore shall a man leave his father and his mother and shall cleave into his wife and they shall be one flesh" (Gen. 2:18, 24) and the other for creation: "Be fruitful and multiply and replenish the earth…." (Gen. 1:28).

The marriage ceremony described in the Bible is referred to simply as "taking a wife" (Deut. 24:1), but from several cases (Jacob, Leah, and Rachel) it can be understood that there were socially defined rules and customs. In the Talmud (the oral law), the marriage ceremony has two parts. The first, kiddushin or erusin (betrothal), took place when the bridegroom gave any object of value (a ring, for example) to the bride and said in front of two witnesses: "With this ring you are consecrated to me according to the law of Moshe and Israel." The second stage took place at a later date (up to a year later) with the marriage proper or nisuin or the Chuppah effected after the bride was brought to the house of the groom and cohabited with him. Today in modern Israel, both the kiddushin and the Chuppah take place at the same event, usually in a wedding hall with the families from both sides and their friends. Different ethnic groups (like Sepharadim or Yemenite Jews) have variations with different traditions.

## LEGAL ASPECTS OF JEWISH MARRIAGE

According to the religious Jewish Law, every man can marry following his "bar mitzvah" (ceremony at the age of 13 years) after which he is qualified and obligated to fulfill all the religious laws. From 0–13 years, he is called a minor (katan) without any legal status, but by 13 years he is called a gadol (an adult). A female is a minor until the age of 12 years, a "naarah" (an adolescent) until 12 and a half years, and only afterwards an adult. From 12 to 12 and a half, she will have to have the permission of her father to marry, but afterwards she is considered an adult.

Child marriage as such in Jewish Law is not a problem as long as the male is 13 years and the female 12 and a half years old, but in modern Israel the law has been amended and a female cannot marry before the age of 17 years of age. A male who marries a female under 17 years of age will be punished by imprisonment, a fine, or both. However, district courts have jurisdiction to permit a marriage to a girl under 17 years when she has had a child or is pregnant by the male or if there are other special circumstances that permit the marriage, provided the girl is not under 16 years of age. Today this is very rare in Israel, however, with the immigration from Yemen or North Africa in the past, several cases took place.

The criterion for validity of a marriage is a minimal level of understanding (called daat kpeutot or the intellectual capacity of a 6-year-old normally developed child) and the comprehension of the act of marriage[8]. The status of a person with ID in Jewish Law is complicated due to a lack of a definition both in the Bible and the Talmud[9]. The Halacha differentiates between people who have developed normally and those defined as deranged or deaf or shotah with a mental capacity disorder and thought process or behavioral process impaired[9]. The deranged can suffer from mental illness, melancholy, brain injury, or diseases of old age or any other reason, but the Halacha does not make a difference between them and does not categorize them according to etiology, but rather according to level of functioning. Deafness was in the same category as deranged because communication was compromised.

Halacha recognizes situations in which a person functions at a level lower than “daat kpeutot”, but is nevertheless capable of understanding the significance of the act of marriage. This possibility was described by Rabbi Raphael Lipman Halperin (the “Oneg YomTov”, Poland, 19th century) and cited by Farbstein[10]:

> “... a man with a speech defect making his words extremely difficult to understand, and even people used to his company do not always understand his speech, and his mind is very weak, and does not even know how to count, he does not understand the meaning of divorce at all, and it never occurred to him to divorce, because never, since his birth, has he known that divorce exists in the world, and he does not even know anything about the Torah. And whatever he does, he does only because he has habitually seen others doing these things...
>
> This man’s acts of marriage are valid acts, because we have seen that he can adopt acts that he regularly sees in his environment; this person has the legal status of one who is intelligent, because when something is explained, it makes sense to him.”

In other words, the Rabbis took into account situations where people with ID may exhibit greater and lesser abilities in different areas of functioning, being very deficient in one domain while being able to understand complex actions in another. Therefore Rabbi Halperin maintained that if the person understood the meaning of being married, even if the person did not understand the ceremonial act of marriage, the act itself would be considered valid. This position has become Halacha or law.

## CHILDREN

Once a Jewish man has passed “bar mitzvah”, he is obliged to fulfill the command “be fruitful and multiply”, but as mentioned above in modern society today, males wait a little longer in order to get married and multiply. In order to fulfill this commandment, both a male and a female child have to be born, so even after seven girls, the commandment has not been fulfilled.

So for a couple where one or both are persons with ID, there are no restrictions on having children according to Jewish Law. Sterilization is another complicated matter, where Jewish Law is against sterilization of men, it does not apply to women[11].

## INTERACTION IN FAMILIES WITH INTELLECTUAL DISABILITY

One study conducted in Israel[12] was concerned with the lives of the families of four couples with ID. The findings were:

1. **The effect of children on the life of the couple** — This small case study[12] found that children had a negative effect on the functioning of the family. In families with one or more children, the level of positive interaction of the couple decreased, as opposed to families without children. In the families with children, there was less positive interaction on the part of the wife towards the husband and more negative responses from husband to wife when she approached him, as opposed to families without children. Interaction between parents and children were more negative in cases where there was positive interaction between the parents. Families without children exhibited a greater number of positive approaches from the wife towards the husband than in families with one child, in which the number of negative approaches and responses of husband and wife was higher. The fathers with ID could not care for their children properly and when the mothers tried to take care of their children and form positive relationships with them, then family functioning was significantly harmed. When the father with ID felt in competition with his child, he reacted with anger and acted negatively towards his wife. The wife with ID must on the one hand care for her children, but on the other she needed to maintain a normal relationship with her husband.
2. **The influence of several children on family life** — In the families with fewer children, there were improved interactions between the couple. The number of children was related to three functions: parent relations, parent/children relations, and financial status. These functions become worse with more children. Parent relations and relations between parents and children were seen to be very clearly different between families with no children and a family with two children or more. No difference was found between families without children or with one child or more in relation to functions relating to finance, housekeeping, social life, community, and individual adaptation to the family.
3. **Concern for the children** — There was a negative connection between concern for the children and between the two parents with ID. Deep concern for the children on the part of the mother resulted in a decrease in family function since the husband opted to stay away from the house as much as possible.
4. **Mental ability for parenthood** — The ability of these couples to look after their children was low. They needed to depend on each other, in a childish manner, and draw much strength from each other. When a child was born to the family, they were unable to provide it with adult and responsible support that parents usually provide. The fathers, before very dependent on their wives, felt rejected and acted aggressively towards their children.
5. **Education worry** — Education of the children (additional classes, contact with the teacher, buying books and other learning equipment) was considerably low. It was difficult for the parents to provide education and knowledge to their children, apart from the day-to-day worry of food and clothes. Parents expressed, more than once, their frustration concerning their inability to educate their children or guide their behavior and turned to the social workers and institutions to accept responsibility for the education of their children.
6. **Dependence on assistance from the environment and institutions** — Assistance provided by the extended family created an important base for success of family life. For families with a bad financial situation and fathers unemployed, assistance was generally provided by the wider family circle or by a support family in close contact with the couple. Sometimes, in extreme circumstances, the children were sent to residential care. This assistance from the wider family circle enabled the mother to be more available for her children. In providing assistance to the mother, the parents, brothers, or supporter decreased slightly the competition between the child and the husband. In this situation, the relations between the father and child increased

> significantly. The family could function better with the assistance of the extended family and various social institutions, but the extended family could not care for the children, who in most cases were cared for by strangers or referred to day care institutions. In the families where the child left the family for residential care, there came a certain amount of calm in the couple's relationship, so that the wife could devote herself to shaping her life and mutual relations with her husband and the husband could become more positive in his reactions and enjoy life again with his wife.

An obvious conclusion drawn from this small sample of four families in Israel pointed to the fact that individuals with ID can marry and live a harmonious and warm life together, but the aspect of children could add a significant factor of negative influence on the function of the family. This situation must therefore be seen as a challenge to social services to provide better support, but also protect the children at the same time.

## DISCUSSION

In this paper, we have discussed the attitude of Jewish Law toward the marriage of persons with ID and found that there is no prevention of such a marriage. They may even have children within the framework of the law. Jewish Law is more liberal than American Law in this regard. The Mental Deficiency Act of 1913, as an example, made marriage illegal for persons with mental retardation everywhere in the U.S.[13]. This has changed and in the future we will see many more cases, even though data are scarce today. It has been estimated[13] that there are approximately 1.4 million parents with ID in the U.S. between the ages of 18 and 64 years with children under 18 years of age.

The main issue involved is the welfare of the children, both when we discuss children of parents with mental illness[14] or ID[15]. Data from the U.S., the U.K., and Australia[13] are beginning to demonstrate that parents with ID are:

- Overrepresented in childcare proceedings
- Less likely to have received support in their parenting
- At greater risk to have their parental responsibility terminated on data that would never hold in a case of nondisabled parents
- Likely to have their competence as parents judged against stricter criteria or harsher standards than other parents
- More likely to have their children removed and their parental rights terminated
- Disadvantaged in the child protection and court process by rules of evidence and procedure
- Less likely to receive support in correcting the conditions leading to termination.

And this even though we have little evidence that having parents with ID will have an adverse effect on the child! Researchers from the Sheffield Department of Sociological Studies[1] studied 30 people (16 men, 14 women), aged 16-42 years of age, who had grown up in a family with one parent (28 cases) with ID (usually the mother, 25 cases) with follow-up in-depth interviews. Of the 30 people, half themselves had learning difficulties, which was more than expected. None of these 30 people had had an easy childhood; 11 admitted to skipping school, 11 had been in trouble with the police (three served time in prison), 2 had attempted suicide, 11 were divorced, 16 had experienced some form of abuse, 7 presented or had overcome mental illness, and 8 suffered chronic illness. The overall findings showed that not all children were the victims of their situation and many demonstrated adaptability in coping with a life full of difficulties. There was not a direct correlation between parenting skills and child outcomes since outcome depended on more that just the parents, and it appeared that the support system had had a positive effect. These adults displayed a close relationship with their parents (especially their mother), which was the heart of their adult identity.

A significant psychological aspect of couples with ID, and especially for the woman, is the need to bear a child. The woman regards a child as an emotional need, not a cognitive one, therefore it is of more meaning than if it was a cognitive desire. For the couple, the pregnancy and birth symbolize a status of normal people; it is almost the only thing in which they can resemble normal people, thus here is a position in which they deal with an event having much decisive and existential meaning. This psychological appearance is also seen in the parents of the couple themselves. There exists an additional appearance of wishful thinking — that if their children with ID marry and bring children into the world, "then everything will be all right" and it will convey a sense of normality and approval that up until then did not exist.

Research workers and therapists working with the population of persons with ID refer to a sociopsychological appearance that they have confronted during their work with couples with ID called the "secondary gain". With marriage and childhood, all of a sudden they are interesting — with support and intervention from the community, the extended family, and social services.

In Jewish law, the "grow and multiply" command also relates to raising children and not just bringing them into the world. As for the phrase "raising children", opinions are divided, but the opinion that rules in the Jewish faith emphasizes the concern for the education of the children, not just their health and physical welfare. So in each case of a couple of persons with ID, the welfare services and providers must ask if the cognitive maturity of the couple enables them to raise their children correctly and understand their needs.

## CONCLUSIONS

Jewish Law has been very progressive regarding the possibility of marriage between persons with ID in contrast to American Law, where this right historically had been denied because of the assumption that the children would be better off not being born or being cared for by others. In the case of such a union resulting in children, although they require *some* supervision, family, friends, and social welfare agencies have scrutinized these families so much they are in constant fear of their child being taken away. There is little information on the number of such cases and an overall dearth of information on the effects on the children, although one recent study from the U.K. has shown a varied picture of resilience and a close, warm relationship later on with the family and especially the mother.

## BIOSKETCHES

**Isack Kandel**, **MA, PhD**, is senior lecturer at the Faculty of Social Sciences, Department of Behavioral Sciences, the Academic College of Judea and Samaria, Ariel. During the period 1985–1993, he served as the director of the Division for Mental Retardation, Ministry of Social Affairs, Jerusalem, Israel. E-mail: Kandeli@aquanet.co.il

**Mohammed Morad, MD**, is a specialist in family medicine, lecturer in family medicine at the National Institute of Child Health and Human Development, Division of Community Health, Ben Gurion University of the Negev, and the medical director of a large area clinic in the city of Beer-Sheva. Publications include those on Bedouin health, health aspects, spiritual health, and aging in persons with intellectual disability, and he is a presenter on topics like health policy and services for disadvantaged at national and international conferences. E-mail: morad62@barak-online.net

**Gideon Vardi, MD, MPH**, is a specialist in pediatrics, child health, and development working at the Zusman Child Development Center, Division of Pediatrics, Faculty of Health Sciences, Ben Gurion University of the Negev in Beer-Sheva, Israel. His interest and several publications are in the field of child health and human development, rehabilitation, intellectual disability, and disability. E-mail: GideonVa@clalit.org.il

**Joav Merrick, MD, DMSc,** is professor of child health and human development affiliated with the Zusman Child Development Center, Division of Pediatrics and Community Health at the Ben Gurion University, Beer-Sheva, Israel; the medical director of the Division for Mental Retardation, Ministry of Social Affairs, Jerusalem; and founder and director of the National Institute of Child Health and Human Development. He has numerous publications in the field of child health and human development, rehabilitation, intellectual disability, disability, health, welfare, abuse, advocacy, quality of life and prevention. Dr. Merrick received the Peter Sabroe Child Award for outstanding work on behalf of Danish Children in 1985 and the International LEGO-Prize ("The Children's Nobel Prize") for an extraordinary contribution towards improvement in child welfare and well being in 1987. E-mail: jmerrick@internet-zahav.net. Website: www.nichd-israel.com
